# Supplementary figures and images for: A genetic screen in macrophages identifies new regulators of IFNγ-inducible MHCII that contribute to T cell activation
Source: eLife. 2021 Nov 8;10:e65110. doi: 10.7554/eLife.65110 (PMC8598162; doi:10.7554/eLife.65110)

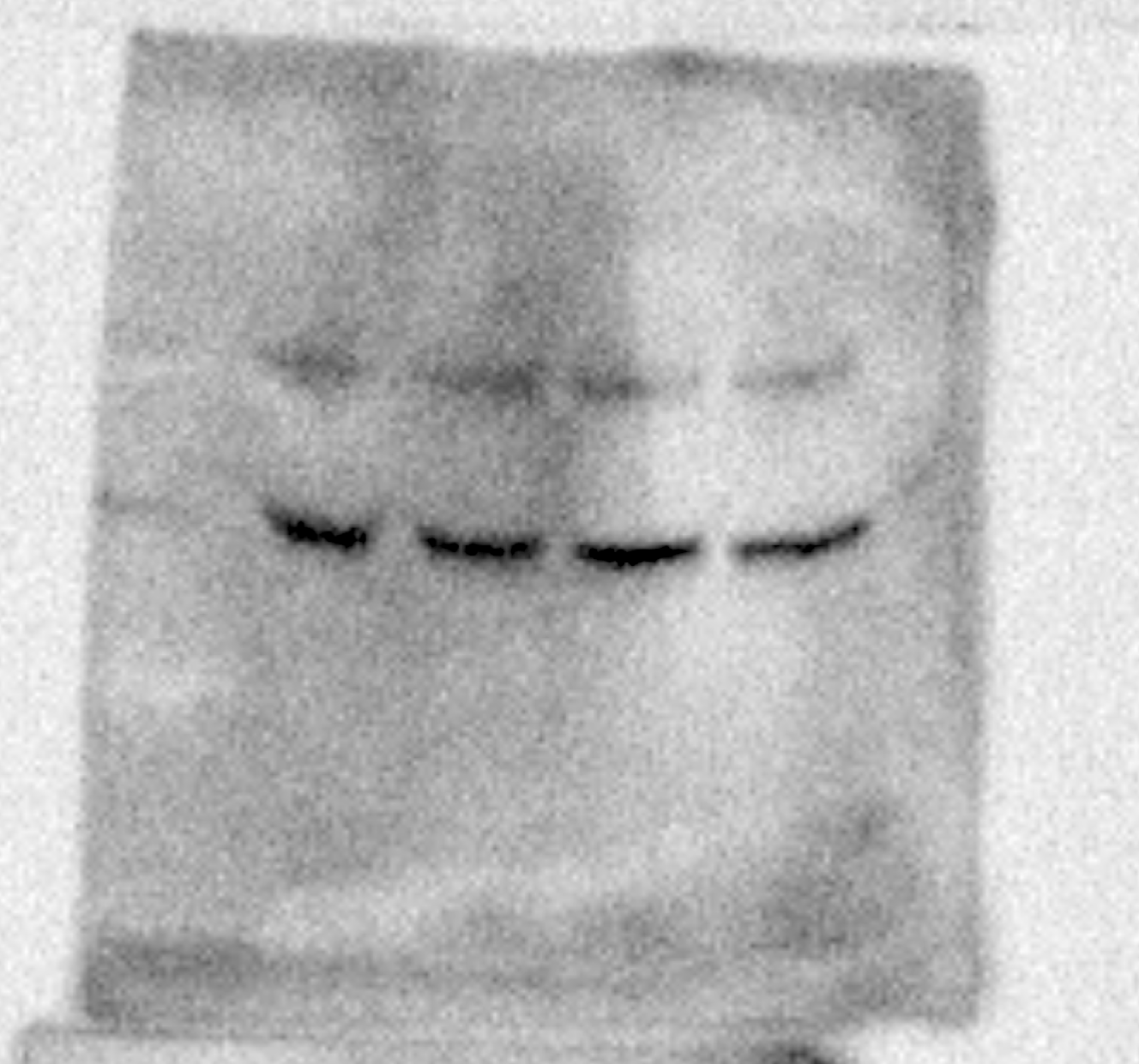

Supplement: Figure 3—source data 1. [file elife-65110-fig3-data1.zip › Figure3_SourceData_1/GSK3_Total_Raw.tiff]

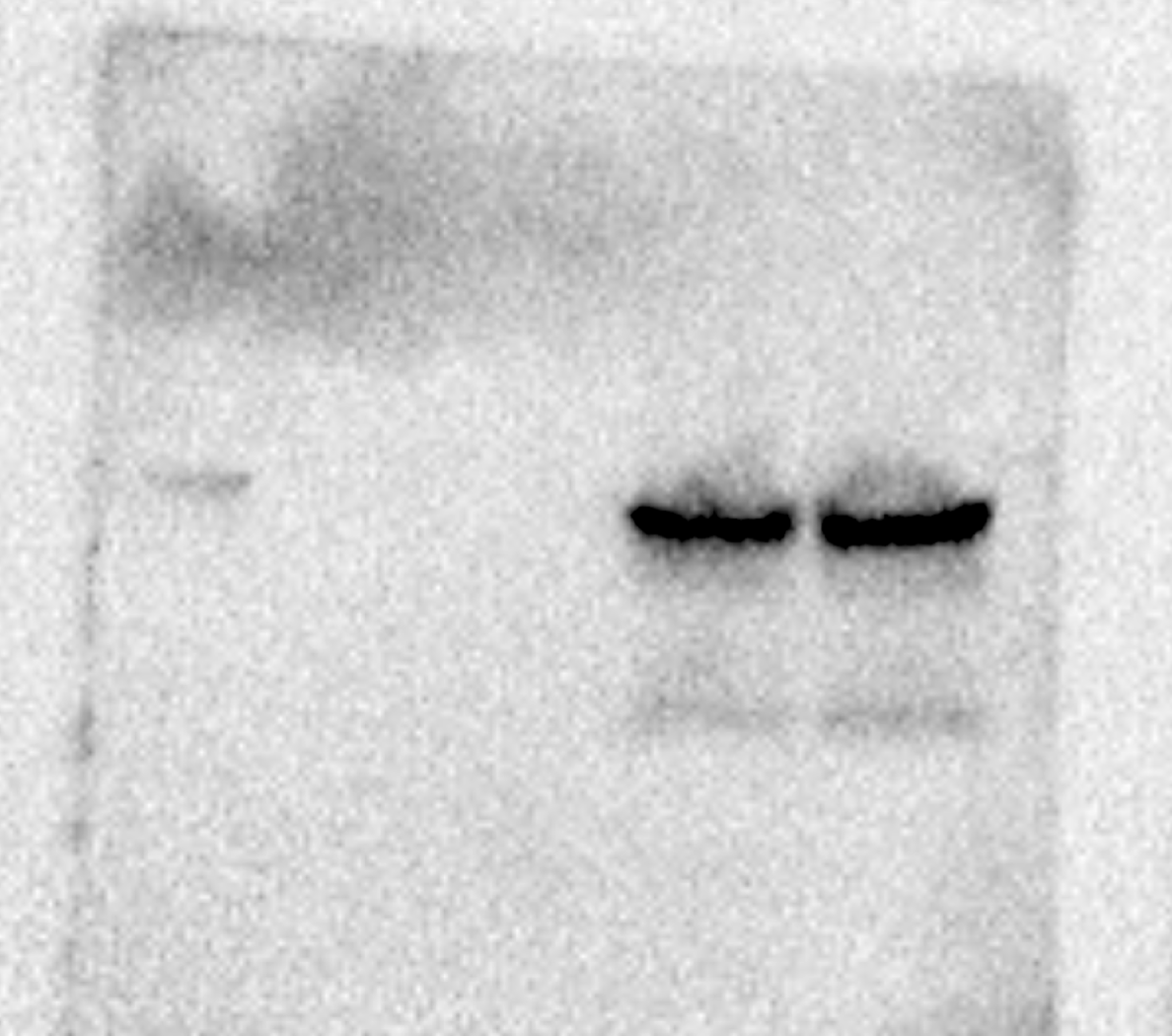

Supplement: Figure 3—source data 1. [file elife-65110-fig3-data1.zip › Figure3_SourceData_1/pStat1_Tyr_Raw.tiff]

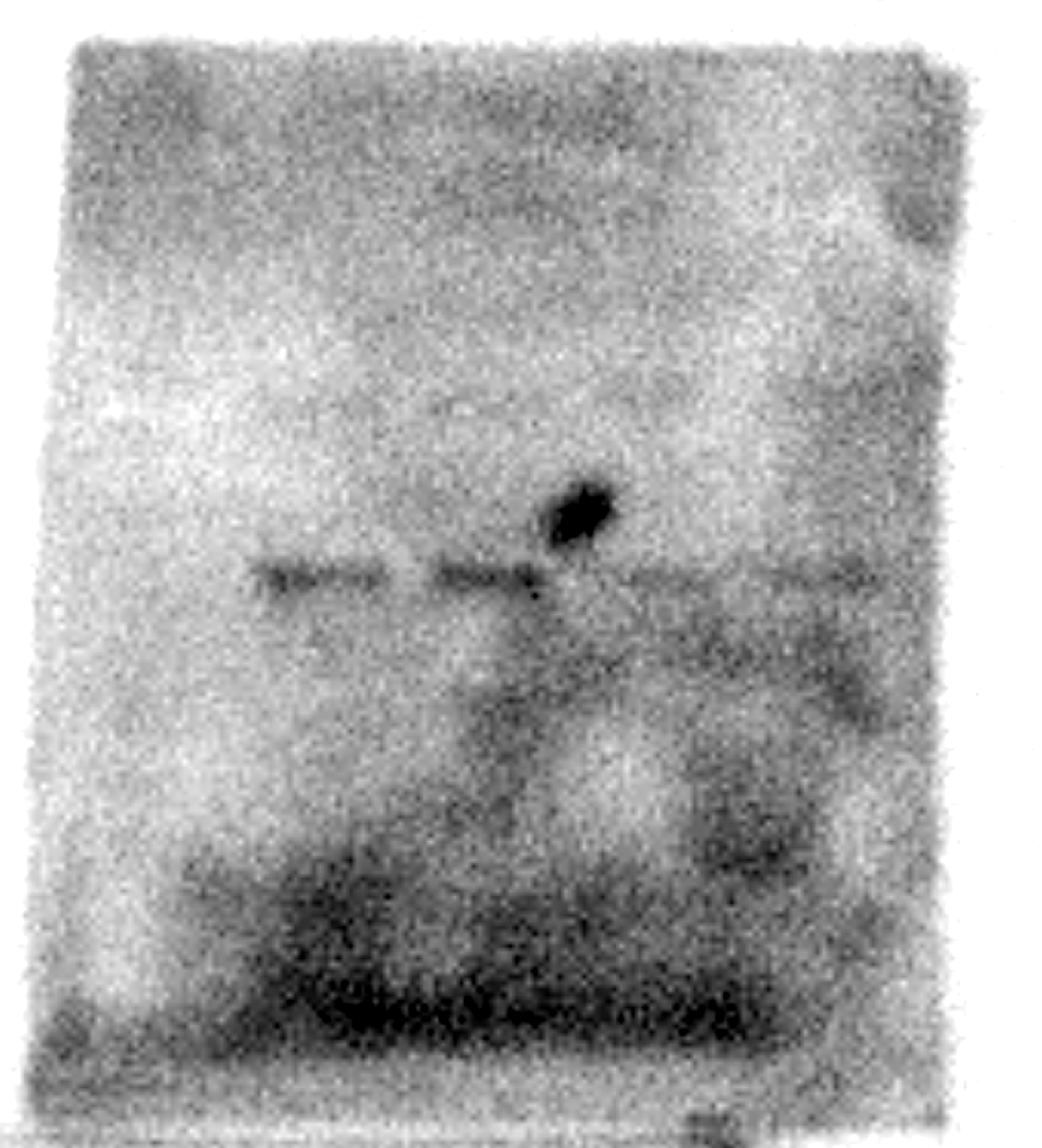

Supplement: Figure 3—source data 1. [file elife-65110-fig3-data1.zip › Figure3_SourceData_1/pGSK3_Raw.tiff]

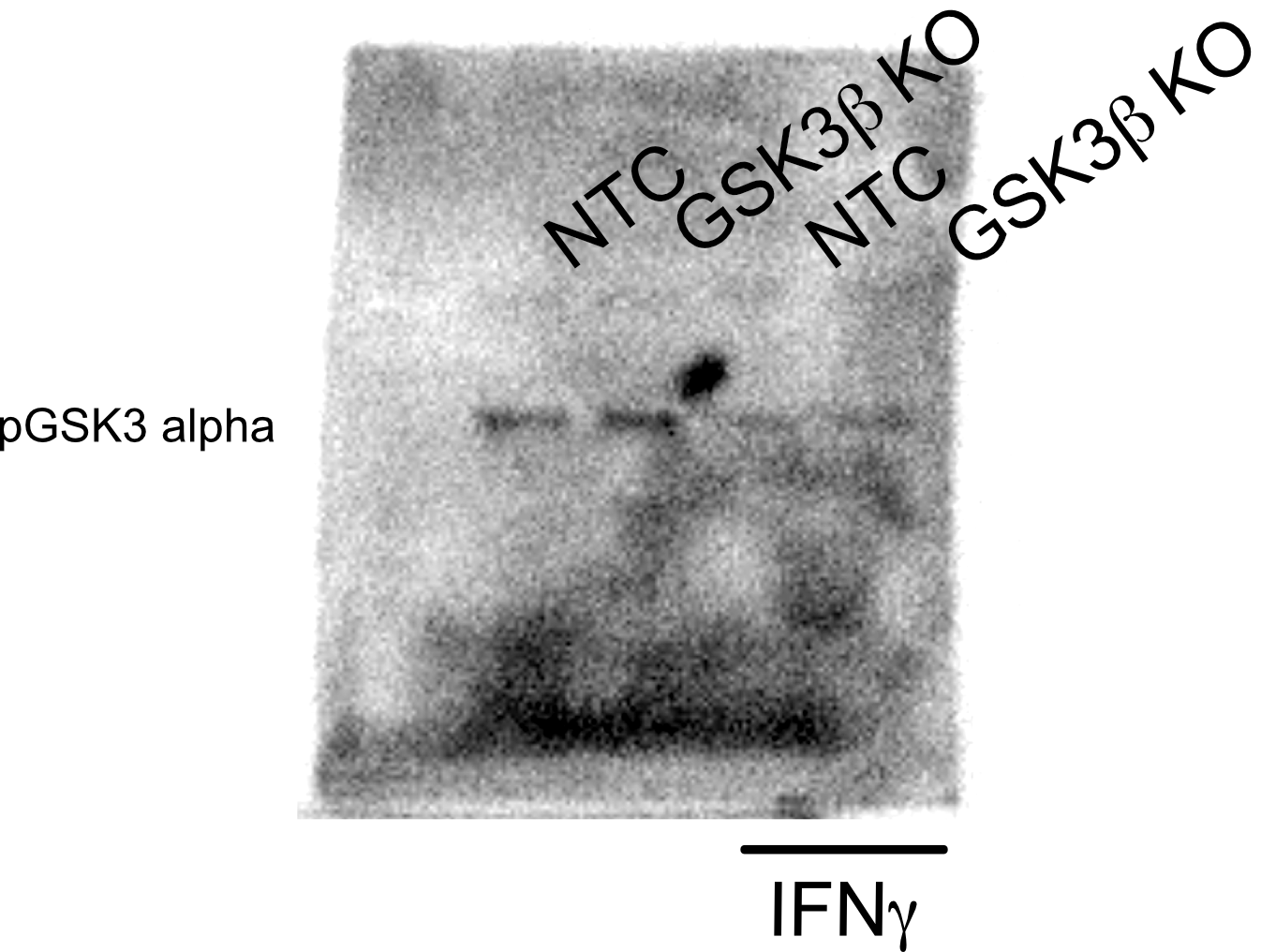

Supplement: Figure 3—source data 2. [file elife-65110-fig3-data2.zip › Figure3_SourceData_2/pGSK3_labeled.tiff]

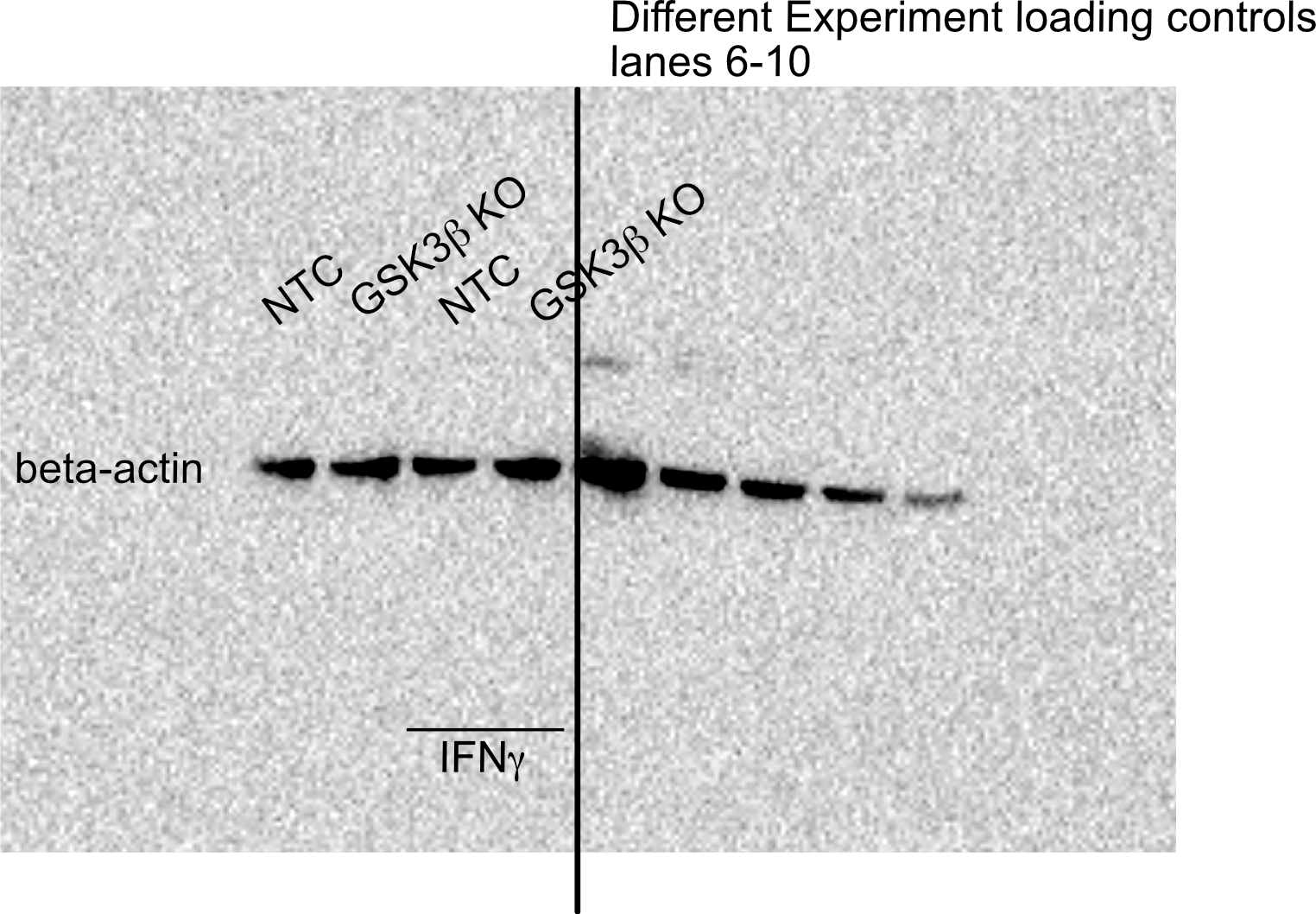

Supplement: Figure 3—source data 2. [file elife-65110-fig3-data2.zip › Figure3_SourceData_2/Actin_labeled.tiff]

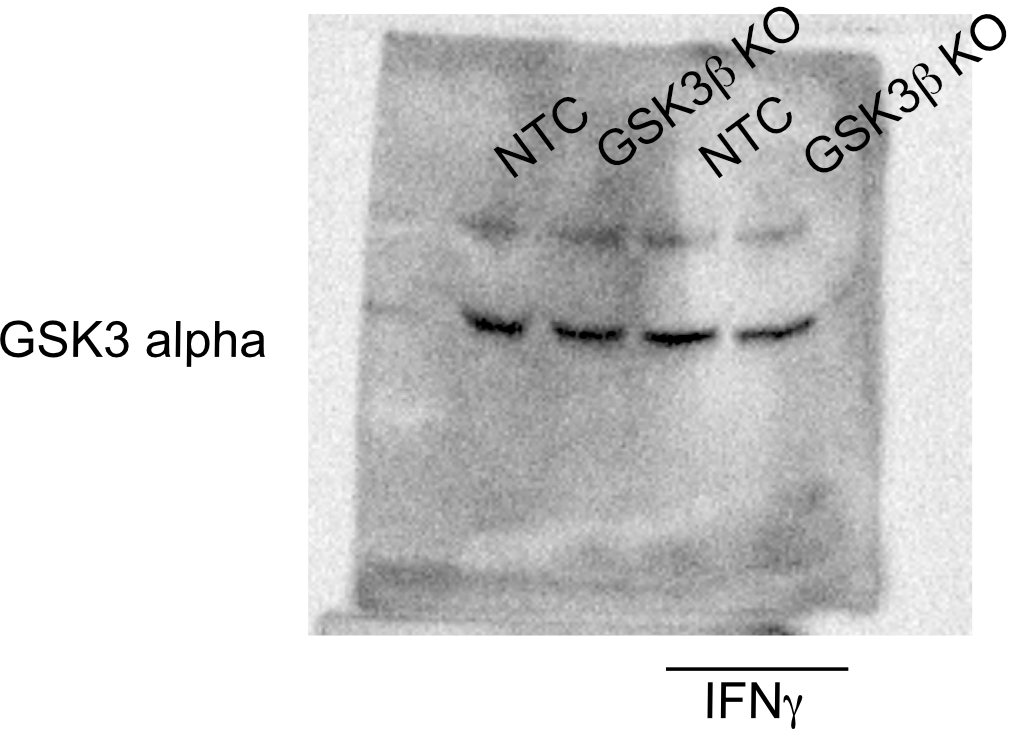

Supplement: Figure 3—source data 2. [file elife-65110-fig3-data2.zip › Figure3_SourceData_2/GSK3_Total_labeled.tiff]

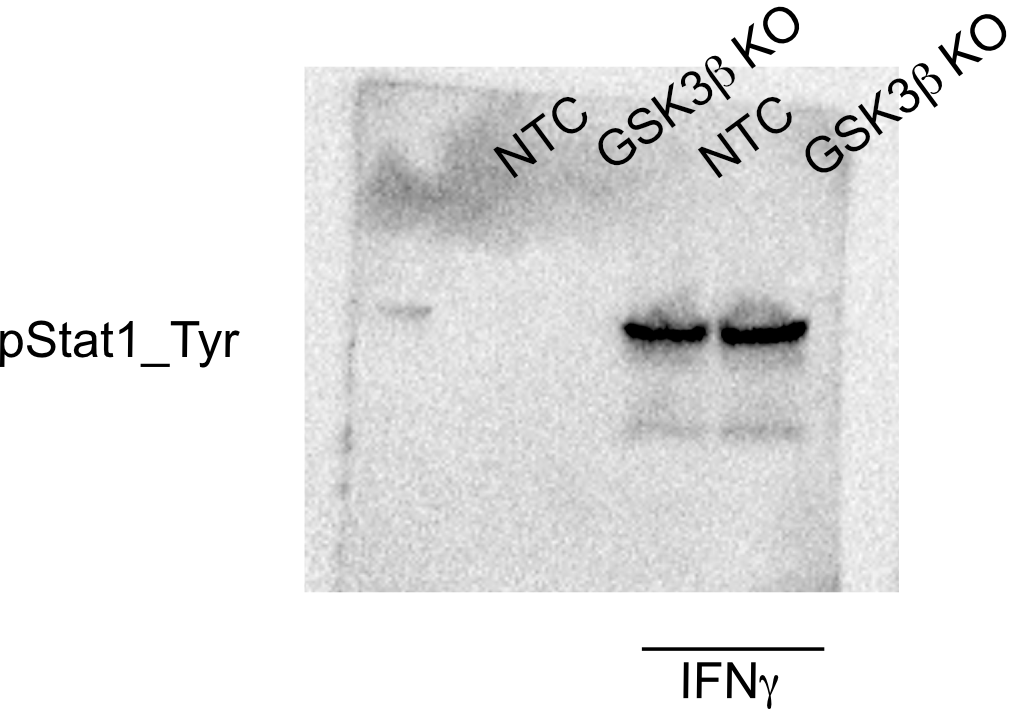

Supplement: Figure 3—source data 2. [file elife-65110-fig3-data2.zip › Figure3_SourceData_2/pStat1_Tyr_labeled.tiff]
